# Safeguarding Vulnerable Autonomy? Situational Vulnerability, the Inherent Jurisdiction, and Insights from Feminist Philosophy

**DOI:** 10.1093/medlaw/fwab010

**Published:** 2021-07-19

**Authors:** Jonathan Lewis

**Affiliations:** Institute of Ethics, Faculty of Humanities and Social Sciences, Dublin City University, Dublin 9, Ireland

**Keywords:** Autonomy, Feminism, Informed consent, Inherent jurisdiction, Liberty, Situational vulnerability

## Abstract

The High Court continues to exercise its inherent jurisdiction to make declarations about interventions into the lives of situationally vulnerable adults *with* mental capacity. In the light of the protective responses of health care providers and the courts to decision-making situations involving capacitous vulnerable adults, this article has two aims. The first is diagnostic. The second is normative. The first aim is to identify the harms to a capacitous vulnerable adult’s autonomy that arise based on the characterisation of situational vulnerability and autonomy as fundamentally opposed concepts or the failure to adequately acknowledge the conceptual relationship between them at common law. The second (normative) aim is to develop an account of *self-authorised, intersubjective autonomy* based on insights from analytic feminist philosophy. This approach not only attempts to capture the autonomy of capacitous vulnerable adults and account for the *necessary* harms to their autonomy that arise from standard common law responses to their situational vulnerability, it is also predicated on the distinctions between mental capacity, informed consent, and autonomy, meaning that it is better placed to fulfil the primary aim of the inherent jurisdiction—to facilitate the autonomy of vulnerable adults with capacity.

## I. INTRODUCTION

Recently, increased legal, ethical, and philosophical attention on different conceptions of vulnerability have paralleled developments concerning the respect for, and protection of, patient autonomy. By responding and adapting to issues concerning problematic conceptualisations of the terms ‘vulnerability’ and ‘autonomy’ in legal contexts, legal scholars and feminist philosophers have called into question the adequacy of common law approaches to autonomy. Additionally, these criticisms have raised substantive problems with the application of standards of rationality and reason at law.

The most important issue concerns the perception of autonomy and situational vulnerability (in the sense of situational risks to an individual’s autonomy of decision making) as oppositional concepts at common law. The courts have been compelled to exercise the inherent jurisdiction to make declarations about interventions into the lives of those deemed to be at risk of constraint, coercion, undue influence, and so on, even when they are deemed to have mental capacity. As Paul Skowron has observed by descriptively teasing out the way in which the concept of autonomy has been used across a range of mental capacity cases, ‘if a person is found to have capacity, then they will be presumed to be autonomous, but that presumption may be rebutted if they are found to be vulnerable and subject to coercion’.^1^ It follows that capacitous vulnerable adults can be denied their decision-making authority not only so that—what the law presumes to be—more rational decisions may be affected, but also to protect them from malign external influences that would otherwise vitiate their consent to medical treatment.

Recent legal scholarship—in dialogue with feminist philosophy—has focused on the broader dimensions of vulnerability, including *ontological* and *pathogenic* forms of vulnerability.^2^ This article is primarily concerned with diagnosing the problems with the two standard common law responses to the *situational* vulnerability of capacitous adults understood in terms of the risks to an individual’s autonomy of decision making. For the sake of clarity, and unless otherwise stated, when the term ‘capacitous vulnerable adults’ is used in this article, vulnerability should be interpreted in its *situational* sense.^3^

There are two reasons for focusing on current legal responses to the situational vulnerability and autonomy of specifically *capacitous* adults. First, from an autonomy perspective, I endorse the distinction between mental capacity and incapacity in principle. The conditions of mental capacity presented in section 3(1) of the Mental Capacity Act (‘MCA’) 2005, for example, are standardly taken by theorists of autonomy to be necessary (though insufficient) conditions for the *capacity* for autonomy.^4^ Thus, in principle, if an individual is *correctly* judged to lack capacity, then they will not be able to satisfactorily exercise their autonomy because they lack the necessary cognitive capacities necessary to do so. Of course, as Beverley Clough and Jaime Lindsey have argued, the lack of capacity should not be straightforwardly assumed based on a person’s intrinsic or inherent vulnerability (ie their physical or intellectual disabilities).^5^ Secondly, Jonathan Herring and Jesse Wall have observed that the MCA 2005 does not sufficiently distinguish between mental capacity and autonomy.^6^ As a result, the Act is unable to deal with cases where individuals, despite being judged to have the capacity and despite their ability to genuinely exercise their autonomy, are judged to be *situationally* vulnerable. Therefore, to capture the autonomy of this specific group of vulnerable individuals, we need to move away from capacity tests and develop a normative framework that can sufficiently delineate mental capacity from the exercise of autonomy. This article will demonstrate, in Sections II and IV, that the primary aim of the High Court’s inherent jurisdiction, which is to facilitate ‘unencumbered decision making’ to support capacitous vulnerable individuals to deliver *genuine* consent, leads to common law responses to situational vulnerability that preclude adequate engagement with the very question of the autonomy (or lack thereof) of capacitous vulnerable individuals. T his is because the fulfilment of the typical conditions for informed consent cannot be equated with the fulfilment of the conditions required for the exercise of autonomy. Consequently, to facilitate autonomy of decision making for vulnerable adults with capacity, the basis on which the inherent jurisdiction is currently used requires reform along autonomy (rather than consent or capacity) lines (see Section V).

The article begins by explaining the standard characterisation of vulnerability at common law (Section II). It also situates the courts’ responses to the situational vulnerability of adults with mental capacity in relation to John Coggon’s and José Miola’s distinction between autonomy and liberty and their analysis of the competing standards that have been used at common law to assess the rationality of decision-making processes.^7^ In the light of recent developments in feminist philosophy, specifically, the branch of feminist theory known as *analytic feminism*, Section III explores how this literature has problematised the two standard common law responses to the situational vulnerability of capacitous patients. It demonstrates how the courts have either perceived the concepts of situational vulnerability and autonomy as conceptually oppositional or failed to adequately acknowledge the conceptual relationship between them. It also articulates the effects on an individual’s autonomy when the courts respond to a capacitous patient’s situational vulnerability based on the perceived conceptual incompatibility between vulnerability and autonomy. In response, certain analytic feminists have argued that autonomy and situational vulnerability are, in fact, *necessarily* entwined concepts. However, as the following Section IV illustrates, the claim that the concepts of autonomy and situational vulnerability are *necessarily* entwined cannot be based solely on mental capacity considerations, standards of informed consent, or processes of ‘unencumbered decision making’ as traditionally used in discussions of vulnerable adults and, more generally, patient autonomy. Furthermore, it will be shown that certain approaches to the concept of ‘relational autonomy’ in analytic feminism also fail to capture the *necessary* harms to the autonomy of capacitous vulnerable adults that result from common law approaches to the conceptual relationship between autonomy and situational vulnerability.

Having diagnosed the problems with common law responses to the situational vulnerability of capacitous adults, Section V develops a reasonable normative framework by which to capture and promote the autonomy of capacitous vulnerable adults. It extends recent analytic feminist scholarship to argue for a particular approach to relational autonomy, one that not only better supports the primary aim of the inherent jurisdiction (to facilitate autonomy of decision making) but also bridges the gap between a patient’s autonomy and their liberty at law (a gap that, according to legal scholars, has proven to be particularly difficult to navigate).^8^ This particular approach to autonomy is used to argue for the duty to promote the autonomy of capacitous vulnerable adults *where possible*, whilst remaining considerate of, and potentially responsive to, more established duties of protection. Consequently, this section presents some general normative considerations by which health care professionals and the courts can navigate the tension between their duty to promote the autonomy of capacitous vulnerable adults and the duty to protect them from harms to their health and well-being in general.

## II. LIBERTY, AUTONOMY, AND THE SITUATIONALLY VULNERABLE ADULT

Informed consent is the standard mechanism through which a patient exercises their liberty at law to reach a decision based on their *sovereignty*—the domain that protects individuals from non-consensual bodily interference.^9^ Violations of a patient’s sovereignty are wrong because they are considered to be trespasses upon the body without explicit, voluntary consent, as opposed to specific interferences with the reasoning processes that govern a patient’s behaviour. Consequently, if there is a domain over which the patient is sovereign, then, based on settled legal principle, lawful reason is required before it is permissible to breach their bodily integrity.^10^ However, juvenility, mental impairment, and factual ignorance all may bar a person from having liberty at law.^11^

Using informed consent as the instrument through which an individual exerts their rightful authority to make a medical decision is problematic because it has led to: (i) the courts confusing the language of autonomy with the concept of liberty and (ii) the running together of the conditions for autonomy and the conditions for mental capacity in the MCA 2005. In terms of the autonomy-liberty distinction, the courts have assumed that if a physician imparts to a patient a list of medically relevant information associated with a treatment and allows the latter to choose based on that information then the patient’s decision is rendered autonomous.^12^ However, such an approach provides no assurances that the patient has, in fact, understood or rationally deliberated on the information with which they have been provided.^13^ Furthermore, with regards to mental capacity and autonomy, although the former is often taken to be a necessary condition for the *capacity for autonomy* to the extent that the latter involves one’s capacities to understand, retain, use, and weigh information relevant to a decision and communicate a decision, the MCA 2005 does not sufficiently distinguish between the conditions for mental capacity and the conditions for autonomous choice and action.^14^ Consequently, neither satisfactory fulfilment of the *capacity* to understand and deliberate, nor the provision of medically relevant information in a way that does not undermine the voluntariness of the decision, is, in themselves or taken together, sufficient to ensure that the resulting decision is autonomous.

To avoid confusing autonomy with liberty, as well as the running together of the conditions for mental capacity and the exercise of autonomy, there have been developments at common law that oblige a physician to ensure that a patient has adequately understood the information with which they have been provided and has reflected on that information in the light of their own values, desires, and motivations in accordance with certain standards of rationality.^15^ Thus, when the statutory test for capacity is interpreted in the light of established medical jurisprudence, ‘there is a concern not just for the capacity for reason, but also for the effective use of it’.^16^ In short, we are required to ‘judge the quality of a person’s exercise of autonomy by the soundness of her reasoning, given her own values’.^17^ Where autonomy (as opposed to liberty or mental capacity) is concerned, English and Welsh medical law demands non-prejudicial deference to the rationality of a patient’s decision and, simultaneously, the values on which their decision is based. Thus, we see in the Supreme Court’s landmark judgment in *Montgomery v Lanarkshire Health Board* [2015] that ‘a patient is entitled to take into account her own values, her own assessment of the comparative merits of giving birth in the “natural” and traditional way and of giving birth by caesarean section, whatever medical opinion may say, alongside the medical evaluation of the risks to herself and her baby’.^18^ Observing that a patient may value one procedure over another, Lady Hale states that ‘the medical profession must respect her choice, unless she lacks the legal capacity to decide’.^19^

One of the reasons for disambiguating between patient autonomy, liberty at law, and mental capacity, is that the former is concerned with the requirement to permit capacitous, legally non-vulnerable individuals to effect changes in their lives in a manner that is consistent with the values, desires, and motivations that they themselves would voluntarily endorse. At the same time, the ‘effective use of reason’ approach to autonomy is needed to identify and respond to concerns regarding the welfare of *vulnerable adults*. As legal scholars have observed, if the law was to ignore a patient’s exercise of their capacity for reason in favour of a purely statutory approach to capacity that supports a patient’s liberty to partake in informed consent,^20^ then this formalised ‘stand-offishness’ would fail to address questions concerning the welfare of those deemed to be *situationally* vulnerable.^21^

According to Robert E. Goodin, to be vulnerable is to be susceptible to threats to one’s interests from particular agents.^22^ Although everyone is potentially vulnerable to such threats, what makes some persons or groups ‘vulnerable’ from the point of view of the law is their dependency on others for care and/or their diminished power to protect themselves from harm or exploitation by others.^23^ Since the introduction of the MCA 2005, there has been debate regarding the High Court’s inherent jurisdiction to make declarations about interventions into the lives of capacitous adults who are *situationally* vulnerable.^24^ Exercises of the inherent jurisdiction are based upon ‘external’ and ‘objective’ assessments of risks to an individual’s power to exercise their autonomy.^^25^^ Although risks can sometimes be identified based on an individual’s characteristics, such as mental impairment or other disability,^26^ legal determinations regarding an individual’s *situational* vulnerability are specifically concerned with identifying the risks of an individual being constrained, coerced, influenced unduly, otherwise ‘incapacitated’ or ‘disabled from giving or expressing a real and genuine consent’ (despite being judged to have the mental capacity to decide).^27^ Furthermore, the High Court recognises that the exercise of the inherent jurisdiction is not necessarily linked to a specific decision that a vulnerable adult is required to make. Indeed, the aim is (often) to prevent circumstances within which an adult might not have the power to make a voluntary decision at an ascertainable point in the future.^28^ Such an approach parallels the public policy ‘safeguarding’ of vulnerable adults from abuse in care services and the statutory protection of vulnerable witnesses in the criminal justice system.^29^

There are two standard common law responses to the situational vulnerability of capacitous individuals. First, the High Court has suggested that it will seek to exercise the inherent jurisdiction to facilitate the process of ‘unencumbered decision making’,^30^ the purpose of which is to ‘allow the individual to be able to regain their autonomy of decision making’.^31^ Such a process attempts to alleviate vulnerability by supporting capacitous vulnerable adults to make decisions free of external pressure or physical restraint,^32^ which would otherwise impact upon their ‘free will and ability and capacity to reach decisions’.^33^ In terms of alleviating vulnerability, ‘the purpose, in respect of a capacitated but vulnerable adult, is to create a situation where he or she can receive outside help free of coercion, to enable him or her to weigh things up and decide freely what he or she wishes to do’.^34^

However, although the courts have stated that ‘unencumbered decision making’ is meant to help situationally vulnerable adults with capacity to ‘regain their autonomy of decision making’ in the face of risks to their autonomy, the *actual* aim—when we take into account the distinctions between autonomy, liberty, and mental capacity as outlined above—is to support a capacitous vulnerable adult to fulfil the typical conditions required for informed consent, thereby, in effect, securing her liberty at law.^35^ Notably, then, the first standard response to situational vulnerability at common law fails, at least in principle, to fulfil its stated aim - to ‘allow the individual to be able to regain their autonomy of decision making’. The reason for this comes down to a lack of appreciation for the specific relationship between the concepts of situational vulnerability and autonomy (discussed further in Section IV below).

The second standard common law response to situational vulnerability implies that the High Court is, at least in principle, concerned with restoring a certain amount of autonomy to a decision-making situation. However, whereas the first standard response attempts to alleviate the vulnerability of the adult through the process of ‘unencumbered decision making’, the second aims to remove vulnerability from the decision-making situation altogether. This is achieved by granting decision-making authority to a designated non-vulnerable third party to make decisions in the best interests of the vulnerable individual in question. As will be demonstrated in Section III below, such a response is predicated on the perceived conceptual incompatibility between the concept of situational vulnerability and the concept of autonomy.

Where questions of autonomy, as opposed to liberty, are specifically concerned, legal scholars have shown that, through the exercise of court powers, the law has developed on the back of two contradictory bases: (i) ‘rational decision-making given an individual’s own values’ and (ii) ‘rational decision-making given some objective or in principle universally acceptable values’.^36^ The former is meant to provide a level of autonomy protection at law for those who are legally *non-vulnerable*. The latter standard is applied to legally vulnerable adults to ensure that ‘more rational’ decisions are effected for the protection of their health or well-being in general.

Where interventions into the lives of situationally vulnerable adults with capacity are concerned, there are two problems tied to the development of two incompatible standards for rational decision making, both of which are based on the two standard common law responses to capacitous adults who are judged to be situationally vulnerable. Where determinations of a vulnerable adult’s ability to make medical decisions for themselves are at stake, the first problem is that the High Court is primarily concerned with the effects of constraint, coercion, or undue influence on their ability to fulfil the typical conditions required for giving *genuine* consent. As Bodey J. observed, determinations regarding a capacitous vulnerable adult’s ability to give or express *genuine* consent should focus *solely* on the effects of malign external influences on the patient’s capacity to manage information relating to ‘proximate medical issues’.^37^ The point is that such determinations should *not* be based on the effects of constraint, coercion, undue influence, and so on, on the soundness of the capacitous vulnerable adult’s reasoning in the light of their own values, desires, and motives. Secondly, and relatedly, the High Court’s focus on the effects of an individual’s situational vulnerability on their ability to exercise their liberty at law precludes adequate engagement with the very question of their autonomy. Specifically, because fulfilling the conditions for *genuine* consent cannot be equated with exercising personal autonomy,^38^ such an approach ignores the possibility that situationally vulnerable adults with capacity can reason soundly in accordance with their own values, desires, and motives and come to a decision that coheres with those motivating reasons, thereby fulfilling the conditions of rational deliberation that philosophers and moral psychologists take to be a necessary feature of autonomy. In response to this problem, Section V offers an alternative normative approach, the employment of which, in principle, satisfies the High Court’s aim to facilitate the autonomy of capacitous vulnerable adults.

## III. ANALYTIC FEMINISM AND ITS RESPONSES TO VULNERABILITY AND AUTONOMY

Like other feminist philosophers, analytic feminists argue that traditional concepts, such as autonomy, rationality, truth, and objectivity, have been ‘perverted’ by androcentrism and sexism throughout the history of philosophy.^39^ However, unlike other feminist approaches, analytic feminists have a ‘core desire’ to retain, and form clear conceptions of, these concepts.^40^ By reproducing philosophical concepts through the application of feminist insights, analytic feminists aim to not only cast new light on issues in philosophy,^41^ but also to generate ‘inclusive’ philosophical theories that ‘work’ for all sorts of women and men, counter sexism and androcentrism, and empower and liberate women.^42^

For this article, one of the discipline’s most important developments has been to challenge the ‘individualistic’ and ‘abstract’ paradigm of the autonomous agent by, first, emphasising the concrete facets of situations in which autonomy capacities are exercised.^43^ Secondly, rather than focus on the dichotomy between the ‘rational individual’ and the ‘social’, analytic feminists have developed arguments that place greater attention on the roles of interpersonal relations, social interaction, and communities in autonomy-determining contexts. This shift of focus from the ‘abstract’ and the ‘individual’ to the ‘concrete’ and the ‘relational’ has informed feminist criticisms of the law’s approach to situational vulnerability.

Even though the ‘effective use of reason’ approach to autonomy is applied at law to protect the welfare of vulnerable individuals, there is a danger, according to analytic feminists, of removing ‘general protections’, such as liberty at law and respect for autonomy, ‘with less clearly agreed or articulated protections’.^44^ It has been argued that this tension between general protections for ‘shared vulnerabilities’ and special considerations for the *situationally* vulnerable can have a number of effects. First, the courts’ focus on the effects of coercion, constraint, and undue influence on a vulnerable adult’s ability to give *genuine* consent, and the primary concern with facilitating ‘unencumbered decision making’, is premised upon the liberal ideal of a free, independent decision or choice, which seems to treat vulnerability as a contingent matter with lawmakers ‘seeking to restore or impose individual responsibility for independence on those who are dependent and vulnerable’.^45^ This kind of facilitative approach too readily discounts how a vulnerable adult’s ability to exercise their autonomy in decision-making situations is dependent on legal and health care recognition.^46^ Second, for those vulnerable adults who are, based on legal definition,^47^ taken to be at risk to threats to their ‘autonomy of decision making’, the denial of decision-making authority and its replacement with substituted decision making or best-interests decisions can compound rather than alleviate such threats.^48^ Third, those who are denied their liberty to partake in decision-making processes that guard against coercion and misinformation may find that they are even more susceptible to malign external influences.^49^ Fourth, analytic feminists have cautioned about the dangers attendant upon labelling particular individuals or groups as vulnerable, arguing that this can lead to discrimination, stereotyping, and objectionably paternalistic social relations and policies.^50^ Fifth, the tendency to focus on a narrow set of duties of protection for the situationally vulnerable largely ignores obligations to promote autonomy wherever possible.^51^

These five criticisms respond to a particular conception of the relationship between autonomy and vulnerability in law and public policy. As Catriona Mackenzie, Wendy Rogers, and Susan Dodds have observed, autonomy and vulnerability can be perceived as oppositional concepts in these two areas. Specifically, they interpret the opposition as a contrast between ‘the liberal (autonomous) subject’ and ‘the vulnerable subject’.^52^ As we have seen, the High Court’s exercise of its inherent jurisdiction is predicated on the distinction between the liberal subject, who is legally recognised as able to give *genuine* consent (without additional support), and the situationally vulnerable subject, who is recognised as unable to give *genuine* consent (without the additional support of ‘unencumbered decision making’) and thereby is denied the opportunity to exercise their liberty at law. For analytic feminists, the opposition between ‘the liberal (autonomous) subject’ and ‘the vulnerable subject’ is problematic for three reasons. First, when it comes to the first standard common law response to situational vulnerability(the facilitation of the process of ‘unencumbered decision making’), ensuring that a situationally vulnerable individual fulfils the typical conditions required for *genuine* consent precludes adequate engagement with the very question of that individual’s autonomy. Secondly, when the courts respond to the situational vulnerability of a capacitous adult by focussing on a narrow set of duties to protect their welfare, the door can be opened to objectionably paternalistic forms of intervention that violate their autonomy.^53^ To understand how these two types of common law response to situational vulnerability violate or preclude adequate engagement with the autonomy of situationally vulnerable adults, it is worth exploring two of the very limited number of cases to deal with questions of health care decision making (as opposed to questions of contact, residence, orsexual consent, for example).

Focusing our discussions on the second standard common law response, the court in *Mazhar v Lord Chancellor* [2017] heard how an NHS Trust had made a without notice, out-of-hours application to the High Court seeking to remove Mr Mazhar, a 26-year-old with muscular dystrophy, from his home and to treat him in hospital.^54^ Mr Mazhar had a tracheostomy and was equipped with a ventilator, with care provided in his home by NHS care staff. He lived with his mother and sisters, who had also been trained to provide specialist care. In all material respects, and, in particular, with regard to decisions about his care, Mr Mazhar was deemed to have mental capacity. However, the Trust made an application to the High Court on the basis that: (i) care staff were not available to tend to Mr Mazhar at his home for one weekend; (ii) his mother was not trained to provide specialist care for him; and (iii) according to a witness statement made by an employee of the Trust, he was oppressively influenced by the forcefully expressed views of a number of his relatives. Based on the evidence available, the judge decided that this was sufficient to make an order under the inherent jurisdiction for Mr Mazhar to be taken to hospital and deprived of his liberty while there. The order was made without Mr Mazhar being notified and without any opportunity to communicate with the court. Furthermore, the order went against Mr Mazhar’s explicit wish not to be taken to hospital.

The case was heard on appeal in October 2020,^55^ during which Baker L.J. stated that although it was unnecessary for the purposes of this case ‘to consider the extent of the inherent jurisdiction in respect of vulnerable adults and, in particular, whether it extends to the making of an order that has the effect of depriving a vulnerable adult of liberty, provided the provisions of article 5 are met’,^56^ the initial judgment was wrong. First, the Trust’s application contained a statement that did not ‘explain whether and, if so, why it was necessary to proceed without proper notice to Mr Mazhar or affording him the opportunity to make representations’—such an explanation was also absent from both the draft and the sealed orders.^57^ Secondly, although Baker L.J. acknowledged that the High Court had jurisdiction to make an interim order in an emergency if the court has ‘reason to believe’ that a vulnerable individual is being unduly influenced, there was nothing in the sealed order to indicate whether the judge had applied this test to Mr Mazhar’s case or on what basis it had been satisfied.^58^ Third, even if the judge had applied this test, Baker L.J. observed that there ‘was manifestly insufficient evidence to satisfy it’.^59^

In one sense, it is obvious how the denial of decision-making authority can violate the autonomy of capacitous vulnerable adults. The point is that the speech acts that Mr Mazhar was denied from performing—refusals—are precisely those speech acts that are otherwise used to deny permissions and exert the boundaries of one’s sovereign authority over one’s body. By denying Mr Mazhar the ability to successfully make refusals regarding the intervention that the NHS Trust deemed to be in his best interest, the court’s exercise of its inherent jurisdiction led to the violation of his autonomy *qua* his sovereignty.

However, as Baker L.J acknowledged, one might argue that this specific violation of Mr Mazhar’s autonomy resulted from a ‘gross and obvious irregularity’ in the application of legal reasoning rather than from the exercise of a jurisdiction that the courts perceive to be ‘substantially protective in nature’.^60^ After all, the judge was not party to the agreed facts and made the order based on the presented evidence. Baker L.J. considered the possibility that the NHS Trust may have believed that the order was an appropriate intervention to the extent that it was made in Mr Mazhar’s best interests given that: ‘(1) Mr Mazhar was in urgent need of specialist medical care; (2) the Trust could not provide that care at home overnight; and (3) on the Trust's case (contested by Mr Mazhar), the family members were not qualified to provide it’.^61^ The problem with this interpretation of the appropriateness of the order is that it ignores the fact that Mr Mazhar was a vulnerable adult *with mental capacity*. In the light of the MCA 2005, such an order would be unlawful if it was made regarding a legally non-vulnerable adult with capacity. Because the judge was aware that Mr Mazhar had capacity in all material respects, it was the Mr Mazhar’s legal status as a vulnerable person that, ultimately, determined the court’s response to the NHS Trust’s application. Specifically, the legal identification of Mr Mazhar as ‘vulnerable’ would have led the judge to question whether Mr Mazhar’s refusal to undertake medical treatment in the hospital could be given voluntarily and whether the undue influence vitiated the refusal. Therefore, because the judge’s response to the case was, in effect, grounded in legal precedent, which, as Baker L.J. acknowledged, had established situational vulnerability in terms of the risks of being constrained, coerced, or otherwise prevented from giving genuine consent, the violation of Mr Mazhar’s autonomy stemmed from the ‘protection imperative’, which tends to ‘arise whenever a court is exercising a jurisdiction that is substantially protective in nature’,^62^ rather than from a problematic application of legal reasoning in this specific instance. Furthermore, this kind of response to situational vulnerability led to the violation of Mr Mazhar’s autonomy because it supported best interests interventions that not only violate a capacitous vulnerable adult’s sovereign authority over their body, but also precluded capacitous vulnerable adults like Mr Mazhar from making claims to autonomy altogether.^63^

Although they are not focused on questions of medical treatment or care, the series of *TZ* cases highlight similar problems with the High Court’s protectionist response.^64^ Even though TZ was declared to have the capacity to consent to sexual relations, there was a concern that, in exercising this capacity in particular instances, he might, in fact, lack capacity as a result of his vulnerability to malign external influences. To resolve this issue, a distinction was made between the capacity to consent to sex and the capacity to consent to contact. As Clough has observed, by drawing such a distinction, ‘the court is entitled to then make best interests decisions on behalf of TZ in relation to particular relationships, as it becomes not a question of sexual capacity, but a point of emphasis on contact’.^65^ However, despite the ability of the court to purportedly make declarations to support TZ to have contact and sexual relations with another individual, the type of support being offered was, ultimately, dependent on what the court deemed to be in his best interests in relation to contact. Thus, according to Clough, ‘support’ is something that can be, in principle, imposed ‘against TZ’s own will and preferences in his best interests’ without adequate consideration of the exercise of his autonomy.^66^

To the extent that interventions based on the perceived conceptual opposition between situational vulnerability and autonomy preclude situationally vulnerable adults with capacity from making claims to autonomy, analytic feminists have suggested that such responses give rise to another form of vulnerability—*pathogenic vulnerability*.^67^ What distinguishes *pathogenic vulnerability* from the concept of vulnerability invoked by the English and Welsh courts is the fact that the former represents the exacerbation of an individual’s already compromised power to exercise their autonomy as engendered by their legal characterisation as someone unable to give genuine consent (due to being at risk, for example, of coercion, constraint, or undue influence). For analytic feminists, the problem with contrasting autonomy and situational vulnerability, and thereby responding to the latter by imposing a protective framework that denies a situationally vulnerable adult their decision-making authority, is that, as we have seen with the cases of Mr Mazhar and TZ, it can have the paradoxical effect of rendering an already vulnerable individual even more powerless to exercise their autonomy.^68^

Turning now to the first standard common law response to situational vulnerability, the case of *A Local Authority v Mrs A and Mr A* [2010] differs from the case involving Mr Mazhar in the sense that Mrs A was deemed to lack the capacity to consent to contraceptive treatment, even though the absence of capacity resulted primarily from the unequal dynamic in the relationship between Mr and Mrs A such that ‘her decision not to continue taking contraception [was] not the product of her own free will’.^69^ The point is that, from an autonomy perspective, Mrs A was deemed to lack mental capacity, the conditions of which are commonly taken to be necessary conditions for the *capacity* for autonomy. Thus, in principle, although it is possible to capture the autonomy of a *capacitous* vulnerable individual by drawing upon the cognitively based procedural conditions commonly taken to be the central features of autonomous choice and action, it is not possible, at least in this specific context, to capture the autonomy of Mrs A using the same theoretical tools (assuming that Mrs A was correctly judged to lack capacity). Instead, as the judge implicitly acknowledged by calling for ‘a capacitated decision from Mrs A’, the first step was to restore her capacity.^70^

Relying heavily on the primary aim of the inherent jurisdiction, the Court of Protection’s preferred outcome was for Mrs A to fulfil the typical conditions required for informed consent. To the extent that these conditions are also the conditions required for mental capacity, restoration of Mrs A’s capacity would, in principle, ensure that she had fulfilled those conditions standardly taken by theorists of autonomy to be necessary (though insufficient) conditions for the *capacity* for autonomy. However, as already acknowledged, fulfilling the typical conditions required for informed consent cannot be equated with the *exercise* of one’s autonomy.^71^ Consequently, at this point, Mrs A^72^ would face the same problems encountered by Mr Mazhar. Specifically, neither the MCA 2005 nor the facilitation of a capacitated decision ‘through “ability appropriate” help and discussion without undue contrary pressure from Mr A’ can capture or promote her autonomy because: (i) the MCA 2005 fails to distinguish between the conditions for mental capacity and the conditions for autonomy and (ii) current applications of the inherent jurisdiction, to the extent that they are concerned with unencumbered decision making, fail to engage with the question of whether a capacitous vulnerable individual fulfils the conditions of *rational deliberation* that philosophers and moral psychologists take to be a necessary feature of autonomous choice and action. In other words, such an approach precludes engagement with the very question of whether Mrs A could reason soundly in accordance with her own values, desires, and motives and come to a decision that coheres with those motivating reasons. Therefore, discounting Mrs A’s lack of capacity, the case reveals the problem with the first standard common law response to vulnerability, that is, the failure of the court to adequately acknowledge the relationship between the concept of autonomy and the concept of vulnerability. More worryingly, as Kirsty Keywood has observed, the judge’s concern with liberty rather than autonomy meant that even if Mrs A could make a capacitated decision having received third-party support, it was by no means clear that Mr A’s malign influence would not have provided grounds for the vitiation of that consent in line with preceding legal responses to the vulnerability of capacitous individuals.^73^

It was previously stated that some analytic feminists take the opposition between ‘the liberal (autonomous) subject’ and ‘the vulnerable subject’ to be problematic for three reasons. The third reason is because the idealised conception of the liberal (autonomous) person fails to attend appropriately to the ways in which an individual’s ability to exercise their autonomy in decision-making situations is dependent on legal and health care recognition.^74^ For example, where liberty is concerned, the practice of informed consent does not just require a capacitous adult patient to communicate a decision having been sufficiently informed of the material treatment risks. Successful participation in an economy of consent depends on the *recognition* of the patient’s speech acts as the kinds of acts that they are, specifically, permissions and refusals.^75^ Where autonomy is concerned, succeeding in making claims to one’s autonomy is impossible for one to do on one’s own (as demonstrated by the cases of Mr Mazhar and TZ). According to Mackenzie, ‘a self-determining life requires not just having the capacities and opportunities to do so but also regarding oneself, and being recognised by others, as having the social *status* of an autonomous agent’.^76^ Thus, although a patient may have the necessary cognitive capacities for reason, their attempts at exercising and achieving autonomy will fail if their commitments, decisions, or status as an autonomous agent are not accorded appropriate recognition. In medical decision-making contexts, succeeding in exercising one’s autonomy is dependent not only on the *recognition* that one has the *status* of autonomy, but also on health care professionals and/or the courts meeting the prescribed uptake conditions associated with one’s permissions and refusals. It follows that when a capacitous vulnerable adult recognises themself as someone with the normative authority to make medical decisions based on their own values, desires, and motives, the denial of that authority not only violates their sovereignty (when medical interventions go against their decisions), it also violates their status of, and claims to, autonomy. Due to the fact that a situationally vulnerable patient’s status of and claims to autonomy, and thereby the desired outcomes of their decisions, are ultimately dependent on relational practices of recognition, some analytic feminists have argued that autonomy is ‘vulnerable’ to the denial of recognition.^77^ On that basis, they have concluded that the concepts of vulnerability and autonomy are *necessarily* entwined rather than opposed. They are *necessarily* entwined because whether a person—legally vulnerable or not—has the *status* of someone with the authority to be self-governing and self-determining, and, accordingly, whether they are able to make claims to, exercise and achieve their autonomy, is, in part, dependent on the actions of others in ways that are outside of the person’s control.

At this point, it should be mentioned that legal scholars have also acknowledged the problems that arise when ‘general protections’ for capacitous individuals are supplanted by ‘special protections’ for legally vulnerable individuals. Indeed, in some cases, these problems have been identified, in part, via engagement with feminist philosophy.^78^ So why is this article returning to analytic feminist approaches to vulnerability and autonomy rather than seeking to develop those responses to vulnerability that have been presented in feminist legal scholarship? Although it is beyond the scope of this article to offer a comprehensive analysis of the relevant legal literature, the return to analytic feminism is needed because key proposals for legal and social policy reform have addressed current legal responses to vulnerable adults *in general* and have thereby not adequately captured or resolved the specific autonomy concerns of *capacitous, situationally* vulnerable adults.^79^ Ultimately, the problem with these proposals is that they are, in part, informed by the suggestion that for those deemed to be capacitous, situational sources of vulnerability will remain undisclosed, meaning that they will be seen as ‘invulnerable’. However, as we have seen in the cases involving Mr Mazhar and TZ, situational sources of vulnerability do inform common law responses in ways that undermine a capacitous vulnerable adult’s ability to exercise their autonomy. Therefore, one cannot reasonably assume that mere capacity is sufficient to protect a vulnerable individual’s autonomy at law. Nevertheless, as will be shown in Section V, some aspects of these respective proposals regarding the common law treatment of vulnerable adults can be usefully appropriated to enhance a normative framework for capturing and promoting their autonomy. Alternatively, where other proposals for legal and social policy reform have been put forward with the potential to impact upon current legal responses to *capacitous* vulnerable adults, they have not adequately considered the claim that the concepts of autonomy and situational vulnerability are *necessarily* entwined.^80^ By not grounding proposals in this conceptual relationship between autonomy and situational vulnerability, the ability of these legal accounts to successfully capture and promote the autonomy and liberty of capacitous vulnerable adults is significantly limited.

By way of an example, Martha Fineman has focused on the concept of *ontological vulnerability* as a ‘universal, inevitable, enduring aspect of the human condition’ to address the limitations of accounts of inequality and injustice in liberal legal theory.^81^ Like analytic feminists, Fineman takes issue with the concept of the liberal ideal of a free, independent, autonomous, rational subject. She argues for a reorientation of legal theory focused on the *vulnerable* subject, one that ‘encompasses a wide range of differing and interdependent abilities over the span of a lifetime’.^82^ Ultimately, as Mackenzie has demonstrated,^83^ the problems with Fineman’s approach are that it does not explicitly deal with other forms of vulnerability, including the situational vulnerability that comes under the High Court’s inherent jurisdiction. It also, more importantly, sets up the vulnerable subject and the autonomous subject as oppositional concepts in much the same way as we find in common law responses to situationally vulnerable adults. As we have seen, when situational vulnerability and autonomy are conceived as fundamentally opposed, duties of protection can be invoked to justify overly paternalistic forms of intervention that violate autonomy and generate forms of *pathogenic vulnerability*. By contrast, as shall be demonstrated in the following sections, if situational vulnerability and autonomy are conceived as necessarily entwined, then we can argue for the promotion of a capacitous vulnerable adult’s autonomy *where possible*, thereby fulfilling the primary purpose of the inherent jurisdiction as it relates to vulnerable adults with mental capacity - to facilitate ‘autonomy of decision making’. However, in arguing for this response to situational vulnerability, autonomy will not be conflated with an individualised, abstract, liberal conception of autonomy, nor will it be conflated with the fulfilment of the typical conditions required for informed consent, which, as we have seen, is the stated aim of the High Court’s first standard response to situational vulnerability. To capture the autonomy of capacitous vulnerable adults, a specific conception of autonomy in analytic feminism will be developed, one that is relational and necessarily dependent on concrete, intersubjective practices of recognition.

Like Fineman, Kay E. Wilson has also considered the deficiencies in the concept of the liberal, autonomous subject.^84^ However, she also acknowledges the problems that can arise when approaches that focus solely on *ontological vulnerability* fail to address the situational vulnerability that leads to the characterisation of certain adults as ‘vulnerable’ at law. Responding to the feminist scholarship of Mackenzie, Rogers, and Dodds, Wilson has explored how three different types of vulnerabilities—inherent, situational, and pathogenic—can and cannot account for the vulnerability of persons with mental impairments in the light of current mental health law.^85^ In much the same way as I have diagnosed the problems with responses to situationally vulnerable capacitous adults at common law, Wilson has identified ‘mental health law as a cause of, rather than the solution to, vulnerability’ in the sense that it is both discriminatory and ‘unnecessarily restrictive of the legal capacity, liberty, and bodily integrity of persons with mental impairments’.^86^ She has also noted three main approaches to legal reform or abolition: (i) the Abolition with Support; (ii) Mental Capacity with Support; and (iii) Support Except Where There is Harm Models. As she has acknowledged:

all three approaches to the abolition or reform of mental health law and some recent case law developments expanding the meaning of “best interests” [ie in light of section 4(4) and section 4(6) of the MCA 2005] are directed towards giving more empowerment and new legal recognition to the subjective wishes of persons with mental impairments and disabilities.^87^

Of the three approaches, Wilson argues for the Mental Capacity with Support Model, which involves the employment of a protective framework if, after receiving support that allows them to consider their options, a person with mental impairment is deemed to lack mental capacity.^88^ Otherwise, persons with mental impairments should be accorded legal recognition with regards to their care and treatment decisions.

Although Wilson appropriates the work of analytic feminists to diagnose the issues surrounding typical responses to vulnerability in mental health law, the problem with her proposal for a Mental Capacity with Support Model is that it does not follow on from the argument made by those same analytic feminists that the concepts of autonomy and situational vulnerability are conceptually entwined. While Wilson has argued that persons with mental impairments should be accorded the same legal recognition as legally non-vulnerable individuals if they are judged to have mental capacity, the problem, as we have already observed, is that even though situationally vulnerable adults are judged to have the capacity, one of the standard responses at common law involves denying them their decision-making authority on the basis that they are deemed unable to give *genuine* consent. In other words, the success of Wilson’s proposal is, ultimately, dependent on common law responses that either consider situational vulnerability and autonomy to be incompatible concepts, or fail to adequately acknowledge the conceptual relationship between them. Thus, as shall be explained further in the following section, by basing her approach solely on mental capacity, Wilson’s proposal is unable to capture the autonomy and liberty of capacitous vulnerable persons, let alone guarantee legitimate legal recognition for situationally vulnerable individuals in concrete decision-making contexts.

## IV. ACCOUNTING FOR THE ENTWINEMENT OF AUTONOMY AND VULNERABILITY

From an analytic feminist perspective that emphasises the ‘concrete’ and ‘relational’ dimensions of autonomy, the argument that the concepts ‘autonomy’ and ‘situational vulnerability’ are necessarily entwined cannot appeal merely to mental capacity, standards of informed consent, or processes of ‘unencumbered decision making’.

The test for incapacity in section 3(1) of the MCA 2005 raises a strong, negative affirmation of autonomy, whereby an individual is unable to make a decision if they are unable to understand, retain, use, or weigh information relevant to a decision or if they are unable to communicate a decision. Similarly, medical ethicists have appealed to the idea of autonomous patients as competent patients, whereby competency tends to require the capacities to comprehend and critically reflect on information, revise beliefs, and make a decision in the light of information. Mental capacity not only grounds traditional approaches to the principle of respect for patient autonomy in medical ethics but, in accordance with common law doctrine, it supports an individual’s legal capacity to partake in processes of informed consent and thereby—at least implicitly—provides them with the legal opportunity to make decisions based on their own values, desires, and motivations.

The issues here are manifold. First, as already observed, a problem with the MCA 2005 is that it fails to distinguish between the conditions for mental capacity and the conditions for the exercise of autonomy. As Herring and Wall have observed,^89^ the exercise of autonomy ‘is the result of the combination of a cognitive process (understanding facts) and an affective process (attributing value to an outcome)’, whereby the affective process requires ‘that a person’s (first-order) desires are accompanied by the (second-order) appropriation of, or identification with, the desires’, and that the motivating attitudes which an individual endorses or rationally responds to are their own (they are *authentic*).^90^ By contrast, under the MCA 2005, capacity is expressed in terms of a capacity for reason; that is, a capacity for understanding relevant information, using it, and weighing it.^91^ Therefore, although the conditions for mental capacity are often taken to be necessary conditions for the *capacity for autonomy*, they do not sufficiently guarantee one’s ability to successfully *exercise one’s autonomy*. Furthermore, a purely capacity-based approach to autonomy cannot adequately address scenarios where the impaired interaction between the affective component and the cognitive component limits or undermines autonomous decision making. Hence, mere recourse to the MCA 2005 is especially problematic in cases concerning *capacitous* vulnerable adults, who are, by definition, already competent, because their judged vulnerability at law precludes any statutory-based engagement with the question of whether they are able to exercise their mental capacity in the light of their own values, desires, and motivations and in accordance with the standards of rationality commonly required by theories of autonomy.^92^

Secondly, appealing to mental capacity and, by extension, the MCA 2005 as the basis for the claim that autonomy and vulnerability are *necessarily* entwined fails, in practice, to capture the autonomy of vulnerable adults with capacity.^93^ As we have seen, even when an individual is judged to have mental capacity in accordance with the standards listed in section 3(1) of the MCA 2005, the consideration of their capacity in the light of their vulnerability at law, specifically, in the light of those risks that are deemed to compromise their ‘autonomy of decision making’, results in the denial of their decision-making authority to ensure that either the standards of informed consent are fulfilled through a facilitative process or decisions in their best interests are affected. Not only do these common law responses to situational vulnerability combined with standards set in the MCA 2005 fail to capture a capacitous vulnerable adult’s autonomy, but analytic feminists have also argued that responding to vulnerability through a protective framework can violate their autonomy as well as undermine it in other ways. It can contribute to the formation of false norms and beliefs or non-authentic values, desires, and preferences.^94^ It can limit the sorts of values, desires, and motives they are able to recognise.^95^ Additionally, it can contribute to a lack of self-respect and self-esteem,^96^ mistrust of their own decisions,^97^ or an inability to recognise their decisions and commitments as meaningful, worthwhile and valuable.^98^ The point is that overly paternalistic approaches to situational vulnerability can causally shape a vulnerable adult’s practical identity and self-understanding (and thereby their values, desires, motivations, and reasoning processes) in a manner beyond their initial control and in ways that undermine their ability to flourish. In such circumstances, even though the situationally vulnerable adult is deemed to be capacitous, and despite appearing to demonstrate ‘effective use of reason’, it has been argued that their power behind whatever reasoning that gives rise to their behaviour has been compromised such that respecting their decisions would not be consistent with respecting their autonomy.^99^

When it comes to accounting for the conceptual entwinement of vulnerability and autonomy, standard approaches to informed consent in medical law and medical ethics are equally problematic. Due to the establishment of the conception of situational vulnerability in *Re SA* as a legal precedent for subsequent judgments involving the exercise of the inherent jurisdiction, the model of informed consent is incompatible with situational vulnerability.^100^ Legally valid consent requires that it be given voluntarily. However, according to Munby J., the inherent jurisdiction can be exercised in relation to a vulnerable adult who is at risk of not being able to exercise a real and genuine decision to consent.^101^ On the basis that legal precedent has established situational vulnerability in terms of the risks of being constrained, coerced, or prevented from ‘forming or expressing a real and genuine consent’, the model of informed consent excludes those who have been legally identified as vulnerable precisely because the voluntariness of their decisions is deemed to be at risk. As Michael Dunn, Isabel Clare, and Anthony Holland have observed, a judgement that a person has the capacity to consent ‘will be considered an inconvenient truth when that person is also judged to be at risk of being constrained, coerced, or unduly influenced’.^102^

Legal scholars, including Herring, Wall, and Clough,^103^ have respectively endorsed the employment of the inherent jurisdiction as a means to overcome the problems with a purely capacity-based approach to the autonomy of vulnerable individuals. However, such endorsements are based on a largely uncritical acceptance of the High Court’s stated aim to facilitate autonomy of decision making via ‘unencumbered decision making’, which, as we have seen, is a highly problematic interpretation of the aim of the inherent jurisdiction given that the High Court is primarily concerned with facilitating the conditions for *genuine* consent. It should be noted that although Clough does not question the High Court’s ability to fulfil its stated aim, based on the terms according to which unencumbered decision making has been defined at common law, she does recognise the lack of clarity surrounding the principles underpinning the inherent jurisdiction.^104^ Thus, she has claimed that ‘there is a legitimate concern that if principles such as a presumption of capacity, the least restrictive alternative, and the protection of unwise decisions, are ignored, then there is a possibility of purportedly supported decisions becoming coercive, rather than empowering’.^105^ The point is that not only is unencumbered decision making, as currently defined at common law, not to be confused with the restoration of autonomy, but also, as Clough has implied, the success of the employment of the inherent jurisdiction falls outside of the control of situationally vulnerable adults. For example, assuming that the goal is for capacitous vulnerable adults to fulfil the standards of informed consent, we have already acknowledged that successful participation in an economy of consent depends on the *recognition* of patients permissions and refusals as those made by individuals who take themselves to have the *status* of autonomy. Thus, the problem with the model of ‘unencumbered decision making’ is that it fails to acknowledge the fact that whether such a process enables, promotes, or, indeed, undermines or violates a situationally vulnerable adult’s ‘autonomy of decision making’ is, in part, dependent on legal and/or health care recognition of their *status* as someone with the authority to make normatively significant judgements regarding their own care and medical treatment.

As a challenge to the idealised conception of the liberal (autonomous) person, analytic feminists have developed ‘relational’ accounts, which highlight the ‘vulnerability’ of personal autonomy. Such accounts are premised on an understanding of interpersonal and social relationships as background conditions for the development, exercise, and achievement of autonomy.^106^ On that basis, relational theorists have argued that some relationships are hostile to autonomy.^107^ Not only can relations of domination, oppression, and exclusion undermine the capacities required for autonomy,^108^ they can also constrain the sorts of values, desires, and motives an individual is able to recognise, and undermine their respect for themselves and their decisions.^109^ Specifically, relational theorists have tended to focus on how interpersonal and social relations affect the *authenticity* conditions for autonomy.^110^ This coincides with the broader focus on autonomy understood as *self-governance*—an individual’s power behind whatever reasoning directly gives rise to their behaviour.^111^

To the extent that relational approaches to autonomy have tended to focus on how interpersonal and social relationships affect the *authenticity* conditions for autonomy, they cannot function as a plausible interpretation of the *necessary* conceptual entwinement of the concepts of situational vulnerability and autonomy. Framing this claim in relation to the second common law response to the situational vulnerability of capacitous adults, we have already noted that overly paternalistic approaches can contribute to non-authentic values, desires, and motives, a lack of self-respect, mistrust of one’s own decisions, and an inability to recognise one’s decisions and commitments as meaningful, worthwhile, and valuable. T hese ‘harms’ to the autonomy of situationally vulnerable adults with capacity are *contingent* rather than *necessary*. What this means is that whether a specific capacitous vulnerable adult experiences these effects to their autonomy will, ultimately, depend on their psychological states and dispositions, which, in part, constitute their practical identity and self-understanding (and thereby determine their values, desires, motivations and reasoning processes). Thus, to the extent that certain analytic feminists have focused on the relational dimensions of self-governance, their approaches are only able to explain how autonomy and situational vulnerability are *contingently* entwined. To account for *necessary* conceptual entwinement of autonomy and situational vulnerability, what needs to be explained is how denials of a capacitous vulnerable adult’s decision-making authority generate harms to their autonomy regardless of their individual characteristics and resiliency to the effects of paternalistic intervention.

Returning to the case of Mr Mazhar, the NHS Trust and the High Court were not interfering with his internal cognitive processes that, in part, determined his ability to self-govern. The paternalistic response to Mr Mazhar’s refusal of treatment in the hospital did not seem to directly affect the power behind whatever reasoning directly gave rise to his behaviour. The fact that Mr Mazhar remained committed to his refusal throughout the appeal process demonstrates that he continued to hold power over his reasoning. Instead, what had been ignored by the health care professionals and the High Court was his *status* as someone with the authority to be self-governing, an act that led to the violation of his autonomy.

As we have already observed, some analytic feminists have been able to explain the harms to autonomy that Mr Mazhar experienced. This has involved the exploration of the relational dimensions of autonomy beyond the effects of interpersonal and social relationships on one’s reasoning processes and the values on which they are based. For these theorists, what accounts for the *necessary* conceptual entwinement of autonomy and situational vulnerability is the fact that autonomy is necessarily dependent on relational practices of recognition.^112^

## V. PROMOTING THE AUTONOMY OF CAPACITOUS VULNERABLE ADULTS AND THE DUTY OF PROTECTION

A capacitous vulnerable patient cannot determine whether the decisions they make regarding their care and treatment will be respected. To succeed, they must, ultimately, be recognised as an individual with the *status* of someone who has the authority to make normatively significant judgements about matters that concern them. This is what Mackenzie has referred to as the ‘self-authorisation’ dimension of autonomy, which ‘involves regarding oneself [and being recognised by others] as having the normative authority to be self-determining and self-governing’.^113^ Accordingly, to regard oneself as having the authority to raise and defend claims to one’s autonomy as a person with equal standing, one must view oneself as a ‘legitimate source of reasons for acting’.^114^ As Axel Honneth and Joel Anderson have observed, ‘if one cannot think of oneself as a competent deliberator and legitimate co-author of decisions, it is hard to see how one can take oneself seriously in one’s own practical reasoning about what to do’.^115^ Such ‘self-respect’ must be *genuine* in the sense that one must be disposed to vouch for the self-recognition of one’s normative authority as *warranted* or *deserved*.^116^ According to analytic feminists who have explored this account of self-authorisation, one’s normative authority should be recognised as warranted or deserved if one is in control of one’s values, desires, and motivations. In short, I must recognise that the values on which I deliberate are *my own* rather than the products of malign external influences.^117^ In turn, analytic feminists have argued that once one takes one’s authority to be legitimate, one accepts that one is able to speak for oneself and thereby answer to others.^118^

To account for the necessary relationship between vulnerability and autonomy, analytic feminists who have adopted the self-authorisation approach to autonomy seemingly invoking what Coggon refers to as ‘best desire autonomy’, whereby a decided upon action ‘reflects a person’s overall desire given his own values, even if this runs contrary to his immediate desire’.^119^ Coggon considers this to be the best approach to autonomy when serious decisions are at stake. This raises an important question about how an individual can discern their own motivating attitudes from those that have been formed by malign external influences. The self-authorisation approach to relational autonomy is predicated on an affective attitude of *genuine* self-respect, which necessarily includes a disposition to answer for the soundness of one’s decisions. However, even when someone views themself with respect, they may, nonetheless, adapt their preferences and desires because of the social conditions in which they live. Nevertheless, the adaption of (first-order) preferences and desires is not problematic provided that it is accompanied by the (second-order) critical reflection about one’s own preferences, desires, goals, and values. Such an approach also explains why this account does not follow calls by Lindsey for the courts to analyse vulnerability from an *embodied* perspective.^120^ The embodied dimension of autonomy is highly contested by theorists of autonomy,^121^ and my account is based on the straightforwardly cognitive and rational dimensions of autonomy that most theorists, medical ethicists and, indeed, legal scholars like Coggon, Miola, Herring, Wall, and Keywood, take to be the central feature of autonomous choice and action.

On the basis that viewing oneself as having legitimate authority to make decisions is a necessary condition of autonomy, my account can be used to bridge the gap between autonomy and liberty in medical law. Specifically, self-authorisation includes the idea that patients have the right to protect their domain of sovereignty by expressing their permissions and refusals.^122^ Consequently, from a normative standpoint, self-authorisation grounds the extension of liberty at law to situationally vulnerable adults with capacity. Even though an individual is situationally vulnerable and, therefore, based on legal definition, deemed unable to give genuine consent according to standards of voluntariness, they regard themself as someone who has the *status* of being an autonomous individual. They recognise that they are competent enough and in control of their values, desires, and motivations. As a result, they recognise that they fulfil the conditions necessary to make legitimate decisions regarding their care and treatment. Ultimately, from an autonomy and a liberty perspective, they take themself to be of equal standing with capacitous patients who the law deems to be non-vulnerable.

Recall that self-authorisation, as well as requiring that one regard oneself as having the authority to be self-determining and self-governing, demands that one be recognised by those to whom claims to autonomy are addressed. After all, as analytic feminists have demonstrated, self-authorisation implies that autonomy is an anathema to insulating oneself from critique.^123^ It follows that ‘vouching for oneself puts one’s claim to respect and esteem into the public domain as open to dispute’.^124^ Accordingly, autonomy is also intersubjective. When a capacitous vulnerable patient expresses their permissions and refusals in relation to specific health care interventions, they are appealing to health care professionals and the courts for recognition of their legitimate authority to make their own decisions regarding their care and treatment in line with their own values, motives, and desires. As Joel Anderson has observed, ‘without intersubjective recognition, the “actuality” of what one is vouching for is left in suspension’.^125^

Having outlined a conception of legitimate, self-authorised autonomy that is *necessarily* dependent on interpersonal recognition, the question remains as to how this approach to autonomy could be applied by health care professionals and the courts not only to overcome the problems with standard common law responses to the situational vulnerability of capacitous adults, but also to deal with the tension between two incompatible obligations: (i) the self-prescribed duty of the courts to promote the autonomy of capacitous vulnerable adults, and (ii) the duty to protect them from harms to their health, well-being and other interests. Unlike standard common law responses to situational vulnerability, an approach to autonomy that is based on self-authorisation does not treat the concepts of situational vulnerability and autonomy as conceptually incompatible. Quite the opposite; it is predicated on the *necessary* entwinement of situational vulnerability and autonomy in the sense that an individual’s autonomy is, in part, necessarily ‘vulnerable’ to the denial of legitimate recognition. As a result, so long as a situationally vulnerable individual with capacity satisfies the conditions of legitimacy that have already been detailed and thereby recognises that they are of equal standing with all other (legally non-vulnerable) capacitous patients, there should be no autonomy-based reasons for treating them any differently to a legally *non-vulnerable* patient. It follows that the guiding principle for health care professionals and the courts is to promote the autonomy of capacitous vulnerable patients *where possible*. Because this principle should guide health care professionals in their responses to vulnerable adults in clinical or care-based decision-making contexts, it is worth noting that if this approach to self-authorised autonomy is successfully used, then, in principle, it should reduce the number of applications for pre-emptive, protective intervention made by health care providers to the courts.

If the guiding principle for health care professionals and the courts is to promote the autonomy of capacitous vulnerable patients where possible, then there are three main normative considerations for navigating the tension between the duty to promote their autonomy and the duty to protect them from other harms. First, if the situationally vulnerable individual chooses not to defer the care or treatment decision to health care professionals or the courts, then, as we have seen, they will need to determine whether they have legitimate normative authority to be self-determining and self-governing and thereby to make specific care or treatment decisions based on *their own* values, desires, and motivations. Thus, they will need to determine that the reasons on which their decision is based are not the results of constraint, coercion, undue influence, and so on. If, however, they are unable to affect an attitude of self-respect necessary to take themself to be a legitimate source of reasons for acting and if they are unable to critically distance themself from any adaptive values and desires, then this is a reasonable basis for overriding the duty to promote their autonomy. In short, it is a reason that counts in favour of the duty to protect them from harms to their health, well-being, and other interests through, for example, an application under, or the exercise of, the inherent jurisdiction. However, even though a protective response may be justified in such instances, at least that response will be derived from considerations of autonomy rather than from considerations of the effects of malign external influences on an individual’s ability to fulfil the typical conditions required for giving *genuine* consent, the focus on which precludes adequate engagement with the very question of that individual’s autonomy.

This first normative consideration is primarily concerned with whether a capacitous vulnerable patient fulfils the ‘first-person’ conditions required for the exercise of autonomy. This involves recognising that the reasons on which their decision is based are not the results of malign external influences. As already observed, self-authorisation need not be problematic so long as any adaptive values, desires, or motivations are accompanied by critical reflection on those attitudes to the degree that the reflection, ultimately, motivates the decision. However, given that capacitous vulnerable individuals are, based on legal definition, deemed to be at risk of malign external influence, health care professionals and the courts may be concerned that the process of self-authorisation is still being influenced by the sources of an individual’s vulnerability such that the authenticity of the motivating attitudes is called into question. This leads to an important epistemic consideration, one recognised by advocates of Shared Decision Making in clinical practice.^126^ In terms of who judges whether an individual is able to exercise their autonomy by effectively using their capacity for reason, it is the patient who is epistemically best placed to identify, endorse, and rationally respond to their values. In spite of any lingering reservations that health care professionals and the courts may have concerning the potential effects of coercion, oppression, and manipulation on the exercise of autonomy, a capacitous vulnerable adult is still in the most authoritative position when it comes to their beliefs regarding their reasons for action. Therefore, in accordance with section 1(4) of the MCA 2005, it is not for the health professional or the courts to determine or question the values underpinning a capacitous vulnerable patient’s decision. Nevertheless, this does not preclude the possibility of responding to an individual’s situational vulnerability in ways that might usefully facilitate and promote their autonomy.

First, the process of taking oneself to be a legitimate source of reasons for acting is fully compatible with supported decision making, particularly when health care professionals, social care professionals, family members, or, indeed, the courts assist a situationally vulnerable adult with overcoming any barriers stopping them from successfully authorising their own values, desires, and motives. Referring to Article 12 of the UN’s Convention on the Rights of Persons with Disabilities (‘UNCRPD’), Clough has also argued that supported decision-making should play a vital role in promoting the autonomy of vulnerable individuals.^127^ If a vulnerable capacitous patient requires support to identify, endorse or rationally respond to their values, desires, and motivations, then this process is akin to—what Wilson has referred to as—the ‘Abolition with Support Model’ for responding to vulnerability.^128^ However, whereas Wilson addressed this model as a *sufficient* means for involving vulnerable adults in decision-making processes that concern them, the approach detailed here treats supported decision making as a single, contingent step in a multi-step process of capturing and promoting the autonomy of capacitous vulnerable adults. The main benefit of a response to situational vulnerability based on self-authorisation and intersubjective recognition is that, like the ‘Abolition with Support Model’, it can alleviate situational and pathogenic forms of vulnerability, but it is not anywhere near as radical. Rather than abolishing mental capacity law altogether, the approach outlined here suggests that legal reform is required. In particular, allowing vulnerable patients to make decisions regarding their care and treatment should be a legal obligation grounded in autonomy, rather than in (shifting) interpretations of the UNCRPD. Additionally, the basis on which the inherent jurisdiction is used should be reformed along autonomy (as opposed to capacity or consent) lines.

Second, whereas the courts have tended to focus ‘on labelling and monitoring the vulnerable adult’, an alternative approach could involve the employment of targeted civil law interventions to *only* restrict the situational cause of an individual’s vulnerability (ie the source of coercion, oppression, manipulation, and so on).^129^ As Lindsey has observed, the form that such interventions take will ultimately depend on the specific features of the situation and the individual characteristics of the situationally vulnerable individual, such that they ‘allow the autonomy of the adult to develop free from oppressive influences…in a way which involves the least risk of creating vulnerability through the intervention’.^130^ By way of an example, Lindsey has suggested that the intervention in *A Local Authority v Mrs A and Mr A* [2010] should have taken the form of a court injunction to prevent Mr A from interfering with Mrs A.^131^ However, she has also recognised that, in other cases, an intervention that bans coercive, oppressive, manipulative, or abusive individuals from contacting their situationally vulnerable partners may risk generating more vulnerability on the part of the situationally vulnerable adult. In such instances, more reasonable sets of restrictions may be called for, including, for example, restricting partners of situationally vulnerable adults from being under the influence of alcohol or from being verbally or physically threatening towards their partners and decision-making support staff.

The common theme is that such interventions should only be directed against the *external* cause of an individual’s situational vulnerability and not against the vulnerable individual themself. As a result, these kinds of intervention differ from the current employment of the inherent jurisdiction, which, as we have seen, either denies situationally vulnerable adults their decision-making authority in favour of best-interest decisions or directs interventions specifically at the vulnerable adult to enable them to fulfil the conditions for *genuine* consent at the expense of targeting the *source* of any impairment. By contrast, targeting the *cause* of an individual’s situational vulnerability would, as Lindsey has observed, involve the least risk of generating pathogenic forms of vulnerability that, as we have seen, can render a situationally vulnerable individual even more powerless to make claims to, and exercise, their autonomy.^132^ Additionally, although such an intervention may generate the same result as current employments of the inherent jurisdiction (a space for unencumbered decision making), the purpose of the intervention is not to facilitate the typical conditions required for informed consent, but to support the individual’s ability to exercise their autonomy, which is both conceptually and pragmatically different from informed consent.^133^ Targeting the situational source of vulnerability might also be supported by a capacitous vulnerable adult’s network of family, friends, and social care support.^134^

In terms of a third possible approach to facilitating autonomy, Lindsey has observed that it would wrong to argue that the law can provide a complete solution to what is a significant social problem.^135^ Clough, for example, has explored the implications of a ‘responsive state’ for the provision of supportive background conditions for autonomy. On the basis that ‘the development and sustained exercise of the capacity for self-determination requires ongoing interpersonal, social and institutional scaffolding which can be thwarted by social domination, oppression and disadvantage’, she has argued that ‘the state has obligations to develop social, political and legal institutions that foster the autonomy of citizens’.^136^

In terms of the second normative consideration, if the situationally vulnerable individual is able to recognise themself as having legitimate authority to make their own decisions regarding their care and treatment, then, bearing in mind that self-authorisation implies that autonomy is an anathema to insulating oneself from critique, they should be *disposed* to answer for that decision.^137^ In other words, they should be disposed to demonstrate that their treatment decision coheres with their own values, motives, or values. Although being disposed to vouch for their legitimate normative authority does not morally require a capacitous vulnerable individual to answer for their decisions, health care professionals may reasonably request them to do so in a particular decision-making instance to ensure—what Coggon and Miola refer to as—the *effective use* of their reasoning and thereby to avoid seeking declarations from the courts. Again, if, in such a situation, a capacitous vulnerable adult is unable or unwilling to demonstrate that their permission or refusal coheres with their motivating attitudes, then there is a reasonable basis for the health care professionals and/or the courts to focus on their duties of protection.

At this point, one might question why capacitous vulnerable adults, in particular, are required to be disposed to answer for their decisions. From a pragmatic perspective, unlike for other capacitous patients (those that are legally non-vulnerable), mere capacity is not sufficient to guarantee a vulnerable adult’s autonomy of decision making at law. Developing a normative framework to capture the autonomy of capacitous vulnerable adults involves moving beyond capacity and considering whether an individual is able to exercise their autonomy by effectively using their capacity for reason to identify with, endorse or rationally respond to their motivating attitudes. Relatedly, and from a theoretical perspective, commitment to the proposed relational conception of autonomy demands that if a patient sees themself as the legitimate source of reasons for action, then it is necessarily the case that they are disposed to answer for those decisions. One’s fulfilment of the conditions for the *effective use* of reason cannot be separated from one’s recognition that one is able to speak for oneself and thereby answer to others. Even though a capacitous vulnerable patient is epistemically best placed to identify, endorse and rationally respond to their values, judgements of coherence, as a standard of moral justification, are judgments that any third party, including professionals and the courts, can arrive at once the patient provides them with their decision and values. Ideally, if we could reform the MCA 2005 along autonomy (rather than capacity or consent) lines, then all capacitous individuals (ie whether legally vulnerable or non-vulnerable) would be required to be disposed to demonstrate the soundness of their reasoning. However, given that there is a gap in the Act through which capacitous vulnerable individuals have fallen, and given that consideration of their autonomy is something that is thereby contingent on responses at common law, the normative framework presented here is primarily concerned with offering health care professionals and the courts a way to consider the autonomy of these individuals to better support the primary aim of the inherent jurisdiction—to facilitate autonomy of decision making.

The third consideration relates to the necessary dependence of a vulnerable patient’s autonomy on the recognition of those to whom their claims to autonomy are addressed. The point is that if they satisfy the criteria associated with the preceding two normative considerations, then their legitimate authority to make their own decisions regarding their care and treatment should be recognised by health care professionals and/or the courts, thereby securing their status as an autonomous individual. Furthermore, if health care professionals and/or the courts recognise that a situationally vulnerable adult has the status of autonomy like any other capacitous patient, then, in keeping with section 1(4) of the MCA 2005, they should be allowed to make their decisions no matter how ‘unwise’ they may seem and ‘no matter how unpalatable they may appear to the public’.^138^ Ultimately, if such recognition is granted, then there is no autonomy-based reason not to respect their decision, including their permissions and refusals regarding specific care or therapeutic interventions.

What we can extrapolate from these three normative considerations is a specified version of the claim that health care professionals and the courts should promote the autonomy of capacitous vulnerable patients *where possible*. Specifically, as these three considerations show, for any normative framework based on the concept of self-authorised autonomy, there is the requirement for health care providers and the courts to provide the opportunity for situationally vulnerable patients (and those performing a supportive role in the decision-making process) to fulfil the aforementioned conditions before any pre-emptive duties of protection are effected. As already implied, some health care professionals may be satisfied to recognise a situationally vulnerable patient as someone with the status of autonomy without requiring them to explicitly vouch for the coherence of their decisions. However, if one of the aims of promoting the autonomy of capacitous vulnerable adults is to avoid legal interventions that currently lead to the denial of decision-making authority and the violation of autonomy, then the attending health professional may reasonably request a situationally vulnerable patient to provide details of their reasons for their permission or refusal to determine that their treatment decision does, in fact, cohere with those values, desires, and motives. Of course, the process of having a situationally vulnerable, yet capacitous, patient answer for the legitimacy of their normative authority and thereby the legitimacy of their resulting decisions, may require greater levels of health care professional and/or court support than would usually be accorded a legally non-vulnerable patient. But just because such a process may require more health care or court resources, more time, and, potentially, more detailed exploration of a patient’s motivating attitudes, this is not a good reason for either health care professionals or the courts to avoid prioritising the promotion of a situationally vulnerable adult’s autonomy. Indeed, this does nothing to undermine the guiding principle implied by the self-authorisation approach to autonomy; specifically, that, *where possible*, a situationally vulnerable patient, who legitimately recognises themself as someone with the status of autonomy, should be given the opportunity to express their decision regarding their care or treatment before any pre-emptive duties of protection are effected.

## VI. CONCLUSION

Standard common law responses to situational vulnerability have failed to grant those the law deems to be vulnerable the same opportunities as legally non-vulnerable individuals to make claims to autonomy. Although such approaches have been defended on the grounds that they protect capacitous vulnerable individuals from envisaged harms and exploitation, this protection comes at the ethical expense of either precluding engagement with the very question of their autonomy or violating their autonomy and their liberty at law. An approach that calls for the promotion of autonomy wherever possible does not demand that capacitous vulnerable patients should always be granted authority to make medical decisions where they concern them. Rather, in the sense that a capacitous vulnerable patient’s recognition of themself as having the status to make medical decisions is both normatively significant and intersubjectively dependent, then a patient who recognises themself in such way should, where possible, be given the opportunity to perform those speech acts that express their choice in line with their own motivating attitudes before health care professionals and the courts decide to focus their response on more established duties of protection.

## CONFLICT OF INTEREST STATEMENT

None declared.

## Footnotes

1 P Skowron, ‘The Relationship Between Autonomy and Adult Mental Capacity in the Law of England and Wales’ (2019) 27(1) Med LR 32–58, 54.

2 For example, M Fineman, ‘The Vulnerable Subject: Anchoring Equality in the Human Condition’ (2008) 20(1) Yale JL & Feminism 1–23; M Fineman, ‘The Vulnerable Subject and the Responsive State’ (2010) 60(2) Emory LJ 251–75; KE Wilson, ‘The Abolition or Reform of Mental Health Law: How Should the Law Recognise and Respond to the Vulnerability of Persons with Mental Impairment?’ (2020) 28(1) Med LR 30–64.

3 For engagement with current legal responses to the situational vulnerability of incapacitous individuals in particular, see B Clough, ‘Vulnerability and Capacity to Consent to Sex—Asking the Right Questions’ (2014) 26(4) CFLQ 371–96; J Lindsey, ‘Developing Vulnerability: A Situational Response to the Abuse of Women with Mental Disabilities’ (2016) 24 Fem LS 295–314; B Clough, ‘Disability and Vulnerability: Challenging the Capacity/Incapacity Binary’ (2017) 16(3) Soc Pol’y Soc’y 469–81.

4 GO Schaefer, G Kahane, and J Savulescu, ‘Autonomy and Enhancement’ (2014) 7 Neuroethics 123–36; J Lewis, ‘Autonomy and the Limits of Cognitive Enhancement’ (2020) 35(1) Bioethics 15–22.

5 Clough, ‘Vulnerability and Capacity to Consent to Sex’ (n 3); Lindsey (n 3); Clough, ‘Disability and Vulnerability’ (n 3).

6 J Herring and J Wall. ‘Autonomy, Capacity and Vulnerable Adults: Filling the Gaps in the Mental Capacity Act’ (2015) 35(4) L Stud 698–719.

7 J Coggon and J Miola, ‘Autonomy, Liberty, and Medical Decision-Making’ (2011) 70(3) Camb LJ 523–47.

8 ibid.

9 J Lewis, ‘Getting Obligations Right: Autonomy and Shared Decision Making’ (2020) 37(1) J Applied Phil 118–40.

10 J Coggon, ‘Mental Capacity Law, Autonomy, and Best Interests: An Argument for Conceptual and Practical Clarity in the Court of Protection’ (2016) 24(3) Med LR 396–414, 405.

11 Lewis (n 9) 121.

12 Coggon and Miola (n 7) 535–36.

13 Similar criticisms of the model of informed consent have been developed within medical ethics. For example, S Dodds, ‘Choice and Control in Feminist Bioethics’ in C Mackenzie and N Stoljar (eds), *Relational Autonomy: Feminist Perspectives on Autonomy, Agency and the Social Self* (Oxford: Oxford University Press 2000) 213–35; O O’Neill, *Autonomy and Trust in Bioethics* (Cambridge University Press 2002); R Kukla, ‘Conscientious Autonomy: Displacing Decisions in Healthcare’ (2005) 35(2) Hastings Center Rep 34–44.

14 Herring and Wall (n 6); Clough (n 3); Clough (n 3).

15 Coggon and Miola (n 7) 537–43. Herring and Wall (n 6) 704.

16 Coggon and Miola (n 7) 528. This coincides with more recent developments in medical ethics where it has been argued that a patient’s autonomy pertains to *exercises* of her capacity for reason. See J Holroyd, ‘Relational Autonomy and Paternalistic Interventions’ (2009) 15 Res Pub 325–26; Schaefer, Kahane and Savulescu (n 4); Lewis, (n 4).

17 Coggon and Miola (n 7) 531.

18 *Montgomery v Lanarkshire Health Board* [2015] UKSC 11, [2015] 1 AC 1430, [115] per Lady Hale.

19 ibid.

20 For example, *Re T (Adult: Refusal of Treatment)* [1992] EWCA Civ 18, [1993] Fam 95, [116]–[117] per Lord Donaldson MR.

21 Coggon and Miola (n 7) 527–28; Clough, ‘Vulnerability and Capacity to Consent to Sex’ (n 3); Herring and Wall (n 6); E Cave, ‘Protecting Patients from Their Bad Decisions: Rebalancing Rights, Relationships, and Risk.’ (2017) 25(4) Med LR 527–553, 546; Clough, ‘Disability and Vulnerability’ (n 3); Skowron (n 1).

22 RE Goodin, *Protecting the Vulnerable: A Reanalysis of Our Social Responsibilities* (University of Chicago Press 1985) 112.

23 Lord Chancellor’s Department, *Who Decides? Making Decisions on Behalf of Mentally Incapacitated Adults* (Stationery Office 1997). Also see *Re SA (Vulnerable Adult with Capacity: Marriage)* [2005] EWHC 2942 (Fam), [2006] 1 FLR 867, [82] per Munby J.

24 M Dunn, I Clare, and A Holland, ‘To Empower or to Protect? Constructing the “Vulnerable Adult” in English Law and Public Policy’ (2008) 28(2) L Stud 234–53, 241; Cave (n 20). The question of whether the inherent jurisdiction has survived so as to protect vulnerable adults who are deemed to have mental capacity has, seemingly, been answered by the decision of the Court of Appeal in *DL v A Local Authority* [2012] EWCA Civ 253, [2012] CPLR 504. Also see *London Borough of Wandsworth v M & Ors* [2017] EWHC 2435 (Fam), [2018] 1 FLR 919; *Southend-on-Sea Borough Council v Meyers* [2019] EWHC 399 (Fam).

25 Dunn, Clare, and Holland (n 2) 241. For a broader exploration of vulnerability, beyond capacitous vulnerable adults and the characterisation of situational vulnerability at common law, see Wilson (n 2).

26 *Re SA (Vulnerable Adult with Capacity: Marriage)* [2005] EWHC 2942 (Fam), [2006] 1 FLR 867, [79] and [82] per Munby J.

27 According to Munby J, such circumstances include ‘the effects of deception, misinformation, physical disability, illness, weakness (physical, mental or moral), tiredness, shock, fatigue, depression, pain or drugs’: ibid at [78]..

28 Dunn, Clare, and Holland (n 23) 236.

29 Although medical ethicists recognise that vulnerability is an ontological condition of all human existence, they have also employed the concept to identify those who are especially vulnerable to threats to their ‘dignity’, ‘rights’, and ‘capacity to live as free, autonomous individual[s]’ as a result of situational features. See, for example, UNESCO, *The Principle of Respect for Human Vulnerability and Personal Integrity: Report of the International Bioethics Committee of UNESCO (IBC)* (UNESCO 2013) 9–16. <https://unesdoc.unesco.org/ark:/48223/pf0000219494> Accessed 8 April 2021.

30 *LBL v RYJ and VJ* [2010] EWHC 2665 (COP) [62] per Macur J.; *DL v A Local Authority* [2012] EWCA Civ 253, [2012] CPLR 504 [67] per McFarlane LJ.

31 *DL v A Local Authority* [2012] EWCA Civ 253, [2012] CPLR 504 [67] per McFarlane LJ; *London Borough of Croydon v KR & Anor* [2019] EWHC 2498 (Fam) [40] per Lieven J.

32 Cave (n 20) 533.

33 *LBL v RYJ and VJ* [2010] EWHC 2665 (COP) at [62]–[63] per Macur J.

34 *A Local Authority v Mrs A and Mr A* [2010] EWHC 1549 (Fam) (COP) at [79] per Bodey J.

35 J Lewis, ‘Capturing and Promoting the Autonomy of Capacitous Vulnerable Adults’ (2020) J Med E (online ahead of print). doi: 10.1136/medethics-2020-106835.

36 Coggon and Miola (n 7) 543.

37 *A Local Authority* (n 34) at [64]. For further discussion, see K Keywood. ‘Safeguarding Reproductive Health? The Inherent Jurisdiction, Contraception and Mental Capacity’ (2011) 19 Med LR 326–333.

38 Coggon and Miola (n 7); Cave (n 21); Lewis (n 35).

39 For example, AE Cudd, ‘Analytic Feminism’ in DM Borchert (ed), *The Encyclopedia of Philosophy Supplement* (Macmillan 1996) 20–21; M Fricker and J Hornsby (eds), *The Cambridge Companion to Feminism in Philosophy* (Cambridge University Press 2000); S Crasnow and A Superson (eds), *Out from the Shadows: Analytical Feminist Contributions to Traditional Philosophy* (Oxford University Press 2012); P Garavaso (ed), *The Bloomsbury Companion to Analytic Feminism* (Bloomsbury 2018); A Garry, ‘Analytic Feminism’ in EN Zalta (ed), *The Stanford Encyclopedia of Philosophy* (2018) <https://plato.stanford.edu/archives/fall2018/entries/femapproach-analytic/> Accessed 8 April 2021.

40 Garry (n 39).

41 AE Cudd, ‘Analytic Feminism’ in E Craig (ed), *Encyclopedia of Philosophy* (Routledge 2005) 157–59.

42 Garry (n 39).

43 P Garavaso, ‘Introduction to Feminist Epistemology’ in P Garavaso (ed), *The Bloomsbury Companion to Analytic Feminism* (Bloomsbury 2018) 171–87; A Roth and P Garavaso, ‘Introduction to Feminist Value Theory’ in P Garavaso (ed), *The Bloomsbury Companion to Analytic Feminism* (Bloomsbury 2018) 355–74.

44 W Rogers, ‘Vulnerability and Bioethics’ in C Mackenzie, W Rogers, and S Dodds (eds), *Vulnerability: New Essays in Ethics and Feminist Philosophy* (Oxford University Press 2014) 60–87, 73.

45 S Dodds, ‘Dependence, Care and Vulnerability’ in C Mackenzie, W Rogers, and S Dodds (eds), *Vulnerability: New Essays in Ethics and Feminist Philosophy* (Oxford University Press 2014) 181–203, 198.

46 ibid.

47 *Re SA* (n 26) at [79] and [82] per Munby J.

48 C Mackenzie, ‘The Importance of Relational Autonomy and Capabilities for an Ethics of Vulnerability’ in C Mackenzie, W Rogers, and S Dodds (eds) *Vulnerability: New Essays in Ethics and Feminist Philosophy* (Oxford University Press 2014) 33–59, 39.

49 Rogers (n 44) 73.

50 S Dodds, ‘Inclusion and Exclusion in Women’s Access to Health and Medicine’ (2008) 1(2) Int’l J Feminist Approaches Bioethics 58–79; F Luna, ‘Elucidating the Concept of Vulnerability: Layers Not Labels’ (2009) 2(1) Int’l J Feminist Approaches Bioethics 121–39; W Rogers, C Mackenzie, and S Dodds, ‘Why Bioethics Needs a Concept of Vulnerability’ (2012) 5(2) Int’l J Feminist Approaches Bioethics 11–38; Dodds (n 45).

51 Mackenzie (n 48); Dodds (n 45).

52 C Mackenzie, W Rogers, and S Dodds, ‘What Is Vulnerability and Why Does It Matter for Moral Theory?’ in C Mackenzie, W Rogers, and S Dodds (eds), *Vulnerability: New Essays in Ethics and Feminist Philosophy* (Oxford University Press 2014) 1–29, 16.

53 For a detailed discussion of how the inherent jurisdiction is set up to yield ‘draconian decisions’ that harm vulnerable adults, see A Pugh, ‘Emergencies and Equivocality Under the Inherent Jurisdiction: *A Local Authority v BF* [2018] EWCA CIV 2962 and *Southend-On-Sea Borough Council v Meyers* [2019] EWHC 399 (FAM)’ (2019) 27(4) Med LR 675–86. Also see Cave (n 21) 541–6.

54 *Mazhar v Lord Chancellor* [2017] EWHC 2536 (Fam), [2018] Fam 257.

55 *Mazhar v Birmingham Community Healthcare Foundation NHS Trust & Ors (Rev 1)* [2020] EWCA Civ 1377, [2020] WLR(D) 579.

56 ibid [52].

57 ibid [64] per Baker LJ.

58 ibid [68] per Baker LJ.

59 ibid [69] per Baker LJ.

60 ibid [71] and [56] per Baker LJ.

61 ibid [67] per Baker LJ.

62 ibid [56] per Baker LJ.

63 J. Anderson, ‘Autonomy and Vulnerability Entwined.’ in C Mackenzie, W Rogers, and S Dodds (eds), *Vulnerability: New Essays in Ethics and Feminist Philosophy* (Oxford University Press 2014) 134–61, 144–5. This point was acknowledged by Baker LJ in the case of Mr Mazhar heard on appeal (*Mazhar* (n 55) at [57]).

64 *A Local Authority v TZ (By his Litigation Friend, the Official Solicitor)* [2013] EWHC 2322 (COP); *A Local Authority v TZ (By His Litigation Friend the Official Solicitor) (No 2)* [2014] EWHC 973 (COP).

65 Clough, ‘Vulnerability and Capacity to Consent to Sex’ (n 3) 388.

66 ibid.

67 The development of the concept of pathogenic vulnerability is built upon Goodin (n 21) 194–201. For a discussion of pathogenic vulnerability of persons with mental impairment, see Wilson (n 2).

68 Mackenzie (n 48) 39. Dodds (n 45) 197–201.

69 *A Local Authority* (n 34) at [73] per Bodey J.

70 ibid [75] per Bodey J.

71 Keywood (n 37) 330 also makes this point in relation to Bodey J.’s judgment.

72 *A Local Authority* (n 34) at [75] per Bodey J.

73 Dodds (n 45) 198.

74 A Westlund, ‘Relational Autonomy and Practical Authority’ in P Garavaso (ed), *The Bloomsbury Companion to Analytic Feminism* (Bloomsbury 2018) 375–93, 381. Some analytic feminists have a long-standing concern for conceptions of consent and refusal as well as the exacerbation of pathogenic vulnerabilities through ‘illocutionary disablement’. See, for example, J Hornsby and R Langton, ‘Free Speech and Illocution’ (1998) 4(1) Leg Theory 21–37; R Langton, *Sexual Solipsism: Philosophical Essays on Pornography and Objectification* (Oxford University Press 2009) 25–64; I Maitra and MK McGowan, ‘Discussion: On Silencing, Rape, and Responsibility’ (2010) 88(1) Aus J Phil 167–72.

75 Mackenzie (n 48) 44.

76 For example, J Anderson, ‘Autonomy and the Authority of Personal Commitments: From Internal Coherence to Social Normativity.’ (2003) 6(2) Phil Explorations 90–108; J Anderson and A Honneth, ‘Autonomy, Vulnerability, Recognition, and Justice’, in J Christman and J Anderson (eds), *Autonomy and the Challenges to Liberalism: New Essays* (Cambridge University Press 2005) 127–49; C Mackenzie, ‘Relational Autonomy, Normative Authority and Perfectionism’ (2008) 39(4) J Soc Phil 512–33; A Westlund, ‘Rethinking Relational Autonomy’ (2009) 24(4) Hypatia 26–69; Anderson (n 63); Mackenzie, Rogers and Dodds (n 52); C Mackenzie, ‘Responding to the Agency Dilemma: Autonomy, Adaptive Preferences, and Internalized Oppression’ in M Oshana (ed), *Personal Autonomy and Social Oppression* (Routledge 2015) 48–67; Westlund (n 75).

77 For example, Fineman, ‘The Vulnerable Subject’ (n 2); Fineman, ‘The Vulnerable Subject and the Responsive State’ (n 2); Clough, ‘Vulnerability and Capacity to Consent to Sex’ (n 3); Lindsey (n 3); Clough, ‘Disability and Vulnerability’ (n 3); Wilson (n 2).

78 For example, Clough, ‘Vulnerability and Capacity to Consent to Sex’ (n 3); Lindsey (n 3); Clough, ‘Disability and Vulnerability’ (n 3).

79 For example, Fineman, ‘The Vulnerable Subject’ (n 2); Fineman, ‘The Vulnerable Subject and the Responsive State’ (n 2); Wilson (n 2). In Section IV, I show how some of Wilson’s ideas can complement my proposal for capturing and promoting the autonomy of capacitous vulnerable adults.

80 Fineman, ‘The Vulnerable Subject’ (n 2) 8.

81 ibid 12.

82 Mackenzie (n 48).

83 Wilson (n 2) 31.

84 ibid 38–47.

85 ibid 31–2.

86 ibid 33.

87 ibid 55–6.

88 For example, L Charland, ‘Cynthia’s dilemma: Consenting to heroin prescription’ (2002) Am J Bioethics 2(2) 37–47; B Foddy & J Savulescu, ‘Addiction and Autonomy: Can Addicted People Consent to the Prescription of Their Drug of Addiction?’ (2006) 20(1) Bioethics 1–15.

89 Keywood (n 37) 329–330; Clough, ‘Vulnerability and Capacity to Consent to Sex’ (n 3) 372; Herring and Wall (n 6) 699–700, 708.

90 Herring and Wall (n 6) 699–700, 708.

91 Keywood (n 37) 329–30.

92 For an explanation of why mental capacity cannot be merely equated with autonomy in general and the autonomy of vulnerable adults in particular, see Skowron’s analysis of the use of the concept of autonomy in mental capacity law (Skowron (n 1)).

93 For example, D Meyers, ‘Personal Autonomy and the Paradox of Feminine Socialization’ (1987) 84 J Phil 619–68; D Meyers, *Self, Society, and Personal Choice* (Columbia University Press 1989); D Meyers, ‘Intersectional Identity and the Authentic self? Opposites Attract!’ in C Mackenzie and N Stoljar (eds), *Relational Autonomy: Feminist Perspectives on Autonomy, Agency and the Social Self* (Oxford University Press 2000) 151–80; M Friedman, *Autonomy, Gender, Politics* (Oxford University Press 2003); J Christman, ‘Relational Autonomy, Liberal Individualism and the Social Constitution of Selves’ (2004) 117(1/2) Phil Stud 143–64; J Christman, *The Politics of PERSONS: Individual autonomy and Socio-Historical Selves* (Cambridge University Press 2009).

94 P Benson, ‘Autonomy and Oppressive Socialization’ (1991) 17 *Social Theory and Practice* 385–408; C Mackenzie and N Stoljar, ‘Autonomy Refigured’ in C Mackenzie and N Stoljar (eds), *Relational Autonomy: Feminist Perspectives on Autonomy, Agency and the Social Self* (Oxford University Press 2000) 3–31.

95 Anderson and Honneth (n 77); P Benson, ‘Feminist Intuitions and the Normative Substance of Autonomy’, in JS Taylor (ed), *Personal Autonomy: New Essays on Personal Autonomy and Its Role in Contemporary Moral Philosophy* (Cambridge University Press 2005) 124–42; Mackenzie, ‘Relational Autonomy, Normative Authority and Perfectionism’ (n 77).

96 T Govier, ‘Self-Trust, Autonomy, and Self-Esteem’ (1993) 8 Hypatia 99–120; C McLeod, *Self-Trust and Reproductive Autonomy* (MIT Press 2002); Mackenzie, ‘Relational Autonomy, Normative Authority and Perfectionism’ (n 77).

97 Mackenzie, ‘Relational Autonomy, Normative Authority and Perfectionism’ (n 77).

98 ibid 518–9.

99 Coggon and Miola (n 7) 527–28.

100 *Re SA* (n 26) at [77].

101 Dunn, Clare and Holland (n 24) 247. Also see Skowron (n 1).

102 Herring and Wall (n 6); Clough, ‘Vulnerability and Capacity to Consent to Sex’ (n 3).

103 Clough, ‘Vulnerability and Capacity to Consent to Sex’ (n 3).

104 ibid 394.

105 Christman, ‘Relational Autonomy, Liberal Individualism’ (n 94) 158.

106 N Stoljar, ‘Feminist Perspectives on Autonomy’, in EN Zalta (ed), *The Stanford Encyclopedia of Philosophy* (2018). <https://plato.stanford.edu/archives/win2018/entries/feminism-autonomy/> Accessed 8 April 2021.

107 For example, Meyers, *Self, Society, and Personal Choice* (n 94); Friedman (n 94); Christman, *The Politics of PERSONS* (n 94).

108 For example, Benson (n 95); Govier (n 97); Mackenzie and Stoljar (n 95); McLeod (n 97); Anderson and Honneth (n 77); Benson (n 96); Mackenzie, ‘Relational Autonomy, Normative Authority and Perfectionism’ (n 77); Mackenzie (n 48).

109 Christman, *The Politics of PERSONS* (n 94).

110 Analytic feminists working on issues in medical ethics have already begun to employ relational approaches to autonomy in order to demonstrate the ways in which—what we might call—*vulnerable autonomy* can be incorporated in, and promoted or undermined by, clinical decision-making practices. See, for example, A Donchin, ‘Autonomy, Interdependence, and Assisted Suicide: Respecting Boundaries/Crossing Lines’ (2000) 14(3) Bioethics 187–204; Dodds, ‘Choice and Control in Feminist Bioethics’ (n 13); A Donchin, ‘Understanding autonomy relationally: Toward a reconfiguration of bioethical principles’ (2001) 26(4) J Med Phil’y 365–86; Mackenzie, ‘Relational Autonomy, Normative Authority and Perfectionism’ (n 77); Lewis (n 9).

111 For example, Anderson, ‘Autonomy and the Authority of Personal Commitments’ (n 77); Anderson and Honneth (n 77); Mackenzie, ‘Relational Autonomy, Normative Authority and Perfectionism’ (n 77); Westlund, ‘Rethinking Relational Autonomy’ (n 77); Anderson, ‘Autonomy and Vulnerability Entwined’ (n 63); Mackenzie, Rogers and Dodds (n 52); Mackenzie, ‘Relational Autonomy, Normative Authority and Perfectionism’ (n 77); Westlund, ‘Relational Autonomy and Practical Authority’(n 75).

112 Mackenzie, ‘Responding to the Agency Dilemma’ (n 77) 55.

113 Anderson and Honneth (n 77) 132.

114 ibid.

115 Anderson (n 63) 144.

116 Westlund, ‘Rethinking Relational Autonomy’ (n 77) 30.

117 For example, Anderson, ‘Autonomy and the Authority of Personal Commitments’ (n 77); P Benson, ‘Taking Ownership: Authority and Voice in Autonomous Agency’, in J Christman and J Anderson (eds), *Autonomy and the Challenges to Liberalism: New essays* (Cambridge University Press 2005) 101–126; Mackenzie, ‘Relational Autonomy, Normative Authority and Perfectionism’ (n 77); Westlund, ‘Rethinking Relational Autonomy’ (n 77); Anderson, ‘Autonomy and Vulnerability Entwined’ (n 63); Westlund, ‘Relational Autonomy and Practical Authority’ (n 75).

118 J Coggon, ‘Varied and Principled Understandings of Autonomy in English Law: Justifiable Inconsistency or Blinkered Moralism?’ (2007) Health Care A 15, 235.

119 Lindsey (n 3).

120 Stoljar (n 107).

121 Westlund (n 75).

122 For example, Anderson, ‘Autonomy and the Authority of Personal Commitments’ (n 77); Benson (n 118); Mackenzie, ‘Relational Autonomy, Normative Authority and Perfectionism’ (n 77); Westlund, ‘Rethinking Relational Autonomy’ (n 77); Anderson, ‘Autonomy and Vulnerability Entwined’ (n 63); Westlund, ‘Relational Autonomy and Practical Authority’ (n 75).

123 Anderson (n 63) 144.

124 ibid 145.

125 Lewis (n 9).

126 Clough, ‘Vulnerability and Capacity to Consent to Sex’ (n 3) 383–6.

127 Wilson (n 2) 48–54.

128 Lindsey (n 3) 309–311.

129 ibid 309.

130 ibid 310.

131 ibid 309–311.

132 Keywood (n 37) 329–330; Clough, ‘Vulnerability and Capacity to Consent to Sex’ (n 3) 372; Herring and Wall (n 6) 699.

133 Clough, ‘Vulnerability and Capacity to Consent to Sex’ (n 3) 391.

134 Lindsey (n 3) 312.

135 Clough, ‘Vulnerability and Capacity to Consent to Sex’ (n 3) 382–3.

136 Westlund, ‘Rethinking Relational Autonomy’ (n 77).

137 Pugh (n 53) 686.

137 Pugh (n 53) 686.

## Notes

This work was supported by the European Commission under Horizon 2020 [EnTIRE ‘Mapping Normative Frameworks for Ethics and Integrity of Research’], grant agreement no. 741782.

